# Efficacy of Intragastric Balloon Treatment: Outcomes and Patient Satisfaction 6 Months After Removal

**DOI:** 10.3390/medicina61040616

**Published:** 2025-03-28

**Authors:** Mehmet Sait Berhuni, Hasan Elkan, Baran Yüksekyayla, Vedat Kaplan

**Affiliations:** Faculty of Medicine, Department of General Surgery, Harran University, Sanliurfa 63200, Türkiye; dr_elkan@hotmail.com (H.E.); brn_yuksekyayla@hotmail.com (B.Y.); vedat_kaplan_@hotmail.com (V.K.)

**Keywords:** eating habits, intragastric balloon, obesity, patient satisfaction

## Abstract

*Background and Objectives*: This study aimed to investigate the weight loss, changes in eating habits, and satisfaction with the intragastric balloon (IGB) procedure in patients undergoing treatment for obesity, with follow-up until 6 months after IGB removal. *Materials and Methods*: This study included patients who underwent the IGB procedure between January 2020 and September 2024 at our clinic and were followed-up for 6 months after IGB removal. Patient records were retrieved from the endoscopy database. Parameters before IGB and during IGB removal were obtained from these records. Upon obtaining verbal consent from patients contacted via their registered phone numbers, their self-reported weights 6 months after IGB removal were recorded, and their body mass index (BMI) was calculated. During the same interview, patients were asked about their eating habits before IGB and 6 months after IGB removal, as well as their satisfaction with the procedure, based on questionnaires used in previous studies. The data collected included patients’ age, sex, weight, BMI, percentages of weight and BMI loss at IGB removal and 6 months after IGB removal, duration of IGB placement, maximum weight lost during IGB placement, time to achieve maximum weight lost, changes in eating habits, and satisfaction levels. *Results*: The mean age of the 62 patients who were included in this study was 33.24 ± 8.71 years, with 37 (59.67%) women and 25 (40.32%) men. The mean weight of the patients before IGB was 91.58 ± 7.04 kg, and the mean BMI was 32.00 ± 4.25 kg/m^2^. The mean duration of IGB placement was 5.83 ± 0.41 months, with a mean maximum weight loss of 14.22 ± 4.70 kg and a mean time to achieve weight loss of 3.91 ± 0.91 months. There was a statistically significant difference between the weight before the IGB and the weight at IGB removal (*p* < 0.001). A significant difference was also noted in the weight before the IGB and 6 months after IGB removal (*p* < 0.001). The comparison of satisfaction levels showed no statistically significant difference in eating habits before the IGB (*p* = 0.384), whereas a significant difference was found in eating habits 6 months after IGB removal (*p* < 0.001). The weight loss differences between the satisfied and dissatisfied patients were significant at IGB removal and 6 months after, although no statistical difference was found in the pre-IGB weights between the satisfied and dissatisfied patients (*p* < 0.001). *Conclusions*: Patients undergoing the IGB procedure for obesity should correct their poor eating habits to maintain successful mid-term weight loss results.

## 1. Introduction

Obesity is increasing at an alarming rate worldwide, especially in developing countries [1,2]. The rise in obesity contributes to a concomitant increase in conditions such as hyperlipidemia, hypertension, diabetes mellitus, cardiovascular diseases, cancer, and depression. The growing prevalence of these obesity-related diseases imposes a significant burden and high costs on healthcare systems, making the prevention and management of obesity critical concerns [1].

The etiology of obesity involves both genetic and environmental factors. Poor eating habits (EHs) are considered a social factor among environmental contributors [3]. These include increased meal frequency, irregular meal times, overeating, and fast eating, all of which lead to higher caloric intake and contribute to obesity [4].

A treatment algorithm for obesity is currently being used in clinical practice. This algorithm begins with calorie restriction and increased physical activity. If these initial steps are insufficient, subsequent options include pharmacotherapy, endoscopic procedures, and surgical interventions [5]. For patients who do not respond to diet, exercise, and medication or who are unsuitable for surgery due to high morbidity and mortality risks or personal hesitations, an alternative treatment option is the endoscopic intragastric balloon (IGB). An IGB can also be used as a bridging therapy for morbidly obese patients prior to surgery [5,6]. The weight loss achieved with an IGB reduces the perioperative morbidity and mortality risks for these patients in the subsequent surgical phase [7].

In the existing literature, the majority of studies on IGB (intragastric balloon) application focus on short-term outcomes, while there is a gap in the literature regarding long-term follow-up of these patients. To our knowledge, few studies have followed these patients long term after balloon removal [2]. Therefore, although the 6-month follow-up period in our study may not be considered long-term, it is important as it includes mid-term outcomes. Therefore, this study aimed to evaluate the weight loss, changes in EHs, and patient satisfaction during the 6 months following the removal of the IGB in patients treated for obesity.

## 2. Methods

### 2.1. Study Design and Patient Population

Patients who underwent IGB treatment at our clinic between January 2020 and September 2024 and were followed up for 6 months after balloon removal were included in this retrospective cross-sectional study. This study was conducted in accordance with the principles of the Declaration of Helsinki. Ethical approval was obtained from the institutional ethics committee (date: 10 June 2024, decision no: 08). Patient records were accessed using the endoscopy database. The medical records of all the identified patients were reviewed. Demographic data, as well as pre-IGB and post-IGB removal parameters, were retrieved from these records. Patients were contacted via their registered phone numbers. After obtaining verbal consent to participate in this study, patients’ weight 6 months after balloon removal was obtained, and their body mass index (BMI) was calculated by dividing the body weight (in kilograms) by the square of height (in meters) (BMI = kg/m^2^) and recorded. During the same call, patients were asked a set of survey questions, adapted from previous studies [2,8], to assess their EHs before IGB placement and 6 months after balloon removal and their satisfaction with the procedure.

The survey questions aimed at determining the EHs included four questions regarding meal frequency, meal regularity, meal duration, and the frequency of overeating episodes per week. Each question was scored between 1 and 3 points. Higher scores indicated worse EHs. To calculate the EH total score (EHTS) for each patient, the scores for the four questions were summed, resulting in a minimum score of 4 and a maximum score of 12 (Table 1).

Additionally, two survey questions were designed to assess patients’ satisfaction with the IGB procedure. Patients were asked whether they had achieved their expected weight loss and whether they would recommend the procedure to others. Patients who answered “yes” to both questions were considered satisfied with the procedure, while those who answered “no” to one or both questions were classified as dissatisfied.

The following variables were recorded: patient age, gender, weight, BMI, percentage of weight and BMI loss at the time of IGB removal and 6 months after removal, duration of IGB placement, maximum weight loss during IGB placement, time to reach maximum weight loss, pre- and post-IGB EHs, and patient satisfaction levels.

The entire cohort of patients included in this study consisted of individuals who were not suitable for surgery based on BMI and chose to undergo intragastric balloon placement.

### 2.2. Exclusion Criteria

Patients under 18 years of age, those who received IGB as a bridging therapy prior to obesity surgery, those who dropped out of the follow-up within the 6-month period following IGB removal, patients who had their IGB removed early due to any mandatory reason, patients who did not wish to participate in the study, and those with incomplete medical data were excluded from this study. Additionally, two cases in which patients presented for IGB removal at the 6-month mark but it was found that the balloon was not in the stomach upon endoscopy were also excluded, as the duration of the balloon’s presence in the stomach and its efficacy could not be determined.

### 2.3. IGB Application and Removal

All the patients included in this study were treated with an air-filled IGB (Heliosphere BAG, Helioscopie, Vienne, France). The procedure was performed by the same surgeon (MSB) under sedation provided by the anesthesia team. During the endoscopic procedure, the stomach was evaluated for any incidental pathologies. The IGB was then placed in the fundus of the stomach. Under visualization, the balloon was inflated with 720 cc of air in all the patients. During the procedure, a single dose of an antiemetic (Trimetobenzamide HCl 200 mg, Sanofi Health Products Limited, Paris, France) and an antispasmodic (Hyoscine-N-butylbromide 20 mg, Deva Holding, Kocaeli, Turkey) was administered intravenously as a routine measure. After a short observation period post-procedure, the patients were discharged. All the patients were instructed to follow a clear liquid diet for 3–4 days. They were then advised to gradually progress to nutrient-rich liquids, soft foods, and eventually, a normal diet.

The IGB removal procedure was performed 6 months later, again under sedation in the endoscopy unit. The balloon was deflated under direct visualization and removed, followed by a short observation period before the patient was discharged from the hospital.

### 2.4. Statistical Analysis

The statistical analyses were conducted using SPSS for Windows, version 25.0 (IBM SPSS Inc., Chicago, IL, USA). The Shapiro–Wilk test was applied to assess the normality of the data distribution. Numerical variables that exhibited a normal distribution were expressed as means ± standard deviations, while those not adhering to a normal distribution were reported as medians with interquartile ranges. The categorical variables were presented as frequencies (n) and percentages (%). The comparison of numerical variables between two groups was carried out employing the paired samples *t*-test for normally distributed data and the Mann–Whitney U test for data not normally distributed. For the normally distributed variables, repeated measures analysis of variance (ANOVA) was performed to examine the changes across the three outcome groups over time, while for the non-normally distributed variables, Friedman’s two-way analysis of variance was used. Post hoc pairwise comparisons within groups were conducted with the Bonferroni correction. All the analyses were performed at a 95% confidence interval, with a two-tailed *p*-value of less than 0.05 considered statistically significant.

## 3. Results

The mean age of the 62 patients included in this study was 33.24 ± 8.71 years. Of the patients, 37 (59.67%) were female and 25 (40.32%) were male. The mean weight of the patients before IGB placement was 91.58 ± 7.04 kg, and the mean BMI was 32.00 (4.25) kg/m^2^. The average duration of IGB placement was 5.83 ± 0.41 months. During the IGB placement period, the mean maximum weight loss achieved was 14.22 ± 4.70 kg, and the mean time to reach maximum weight loss was 3.91 ± 0.91 months. The mean EHTS was 8.14 ± 1.14 before IGB placement, compared to 7.45 ± 1.26 at 6 months after IGB removal. Out of the 62 patients, 28 (45.16%) reported being satisfied with the procedure.

### 3.1. Weight Loss

When considering all the patients, the mean weight before IGB placement was 91.58 ± 7.04 kg, which decreased to 79.16 ± 6.72 kg at the time of IGB removal and was 80.08 ± 7.84 kg at 6 months after removal. A statistically significant difference was found between the pre-IGB weight and the weight at the time of IGB removal (*p* < 0.001). A statistically significant difference was also observed when comparing the pre-IGB weight with the weight 6 months after IGB removal (*p* < 0.001) (Table 2). However, no significant difference was observed between the weight at the time of IGB removal and the weight 6 months after IGB removal (*p* = 0.090).

The measurements obtained before IGB placement and at the time of IGB removal showed that the patients lost an average of 13.51 ± 4.21% of their initial weight during the IGB treatment period. When the patient weight before IGB placement was compared with that 6 months after IGB removal, it was found that the patients lost an average of 12.43 ± 5.40% of their initial weight.

### 3.2. BMI Loss

The median BMI of the patients included in this study was 32.00 (4.25) kg/m^2^ before IGB placement, which decreased to 28.00 (3.00) kg/m^2^ at the time of IGB removal and was measured as 28.50 (4.25) kg/m^2^ at six months after removal (Table 2). When comparing the BMI values before IGB placement and 6 months after IGB removal, patients were found to have lost an average of 13.33 ± 4.64% of their BMI.

A significant difference was observed between the pre-IGB BMI and the BMI at the time of IGB removal (*p* < 0.001) (Table 2). Similarly, a statistically significant difference was present between the pre-IGB BMI and the BMI 6 months after IGB removal (*p* < 0.001) (Table 2). However, no significant difference was observed between the BMI at the time of IGB removal and at 6 months after IGB removal (*p* = 0.369).

### 3.3. EHs

Among all the patients, the mean EHTS was 8.14 ± 1.14 before IGB placement and 7.45 ± 1.26 6 months after IGB removal. For the patients who were satisfied with the procedure, the pre-IGB EHTS was 8.28 ± 1.18, which decreased to 6.82 ± 0.90 at 6 months after IGB removal, showing a statistically significant difference (*p* < 0.001) (Table 3). For the patients who were dissatisfied with the procedure, the mean EHTS was 8.02 ± 1.11 before IGB placement and 7.97 ± 1.29 6 months after IGB removal, with no statistically significant change (*p* = 0.396). It was observed that the patients who improved their poor EHs had higher levels of satisfaction with the procedure.

**Figure 1:**
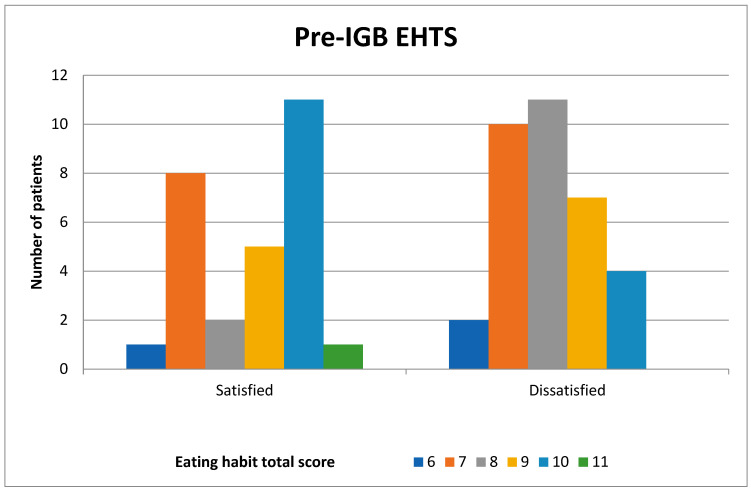
Comparison of the patients who were satisfied and dissatisfied with the procedure in terms of their EHTS before IGB placement (IGB: intragastric balloon, EHTS: eating habit total score).

**Figure 2:**
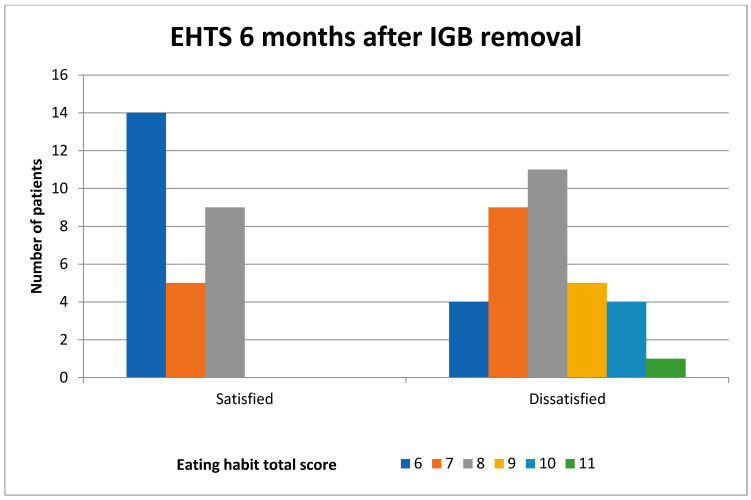
Comparison of the patients who were satisfied and dissatisfied with the procedure in terms of their EHTS months after IGB removal (IGB: intragastric balloon, EHTS: eating habit total score).

When those who were satisfied and dissatisfied with the procedure were compared in terms of their EHs, there was no statistically significant difference between the mean EHTS before IGB placement (*p* = 0.384), but there was a significant difference in the mean EHTS 6 months after IGB removal (*p* < 0.001) (Table 3). It was observed that the patients who improved their poor EHs had their expectations met, resulting in higher satisfaction levels (Figure 1 and Figure 2).

### 3.4. Patient Satisfaction

Of the 62 patients included in this study, 28 (45.16%) were satisfied with the procedure, while 34 (54.84%) were not satisfied. There was no significant difference in the age before IGB placement between those who were satisfied with the procedure and those who were not (*p* = 0.911). Additionally, there was no statistically significant difference in the gender distribution between the two groups (*p* = 0.233). The groups did not show any statistically significant differences in terms of the pre-IGB weight or BMI (both *p* > 0.05).

**Figure 3:**
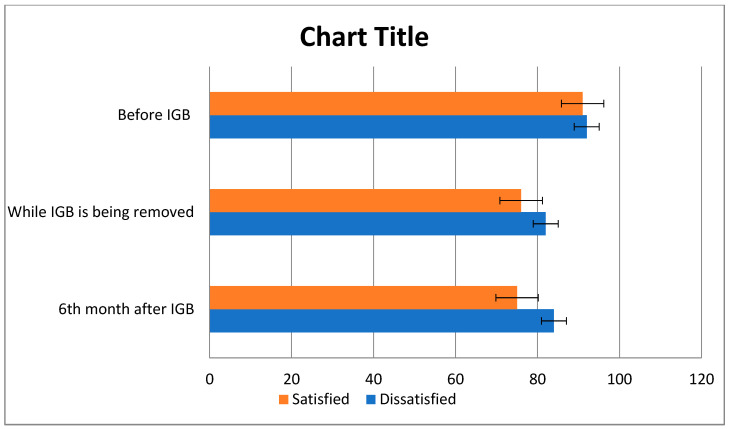
Mean weights before IGB placement, during IGB removal, and 6 months after IGB removal for the patients who were satisfied and dissatisfied with the procedure (IGB: intragastric balloon, kg: kilogram, data are presented as mean ± standard deviation).

The weights of those who were satisfied and dissatisfied with the procedure before IGB placement, immediately after IGB removal, and 6 months after IGB removal are presented in Figure 3. While there was no statistically significant difference in the pre-IGB weight between the two groups, significant weight differences were observed at the time of IGB removal and 6 months after IGB removal (*p* < 0.001). The percentage of weight loss calculated based on the weight values measured before IGB placement and 6 months after IGB removal was 17.28 ± 3.28% in the satisfied group and 8.44 ± 3.01% in the dissatisfied group. There was a statistically significant difference between the two groups (*p* < 0.001).

In terms of the duration of IGB treatment, there was no statistically significant difference between those who were satisfied with the procedure and those who were not (*p* = 0.752). However, the satisfied group experienced significantly greater weight loss during the IGB treatment period (*p* < 0.001), and the time to reach maximum weight loss was significantly shorter in the satisfied group (*p* = 0.002) (Table 4).

## 4. Discussion

In people living with obesity, even a weight loss of 5–10% of the total body weight has beneficial effects on obesity-related health problems [9]. Achieving such weight loss through lifestyle changes alone is often impossible [10]. Although bariatric surgery can lead to significant weight loss, its invasive nature carries risks of morbidity and mortality, and it is not suitable for patients with all BMI levels. At this point, an IGB can be considered an alternative patient treatment method. An IGB may be preferred due to its minimally invasive nature and the minimal side effects, such as nausea and vomiting, which can be controlled with medical treatments within a few days [11].

The primary expectation from IGB treatment is weight loss, with a target of 10–15% total body weight reduction following the treatment [12,13]. Several studies reported the total body weight loss in their IGB patients. Haddad et al. [14] reported a rate of 11.9%, Jerez et al. [15] 11.1%, and Bawahab et al. [16] reported a rate of 14.7%. Similarly, in the present study, this figure was calculated as 13.5% at the time of IGB removal. In the literature, there are only a limited number of publications evaluating the follow-up of patients after IGB removal in the months and years following the procedure [2,14,17]. In a study conducted by Pak et al. in Korean women who underwent IGB treatment, they reported a 12.22% total body weight loss at 6 months after IGB removal compared to the pre-IGB weight [2]. Jense et al. reported an 11% total body weight loss in patients who received 12 months of life coaching after IGB treatment [17]. Similarly, in the present study, 6 months after IGB removal, the total body weight loss was found to be 12.43%. Although one of the studies was conducted exclusively in women, and the other presented 12-month data with additional life coaching, which did not fully align with our study, the total body weight loss figures were comparable. The similarity between our 6-month and Jense et al.’s 12-month data might be because weight regain typically occurred within the first 6 months after IGB removal, or it could be attributed to the prolonged life coaching support in Jense et al.’s study.

Many studies have addressed the issue of poor EHs in people living with obesity [18,19,20]. These publications state that poor EHs include frequent meals, irregular meal times, fast eating (within 10 min), and overeating 3–4 times per week [2,4,19]. A recent study from Brazil demonstrated a significant relationship between irregular meal habits and obesity [20]. In the present study, the patients who managed to maintain their weight 6 months after the procedure, following significant weight loss, had made significant changes in their poor EHs. In contrast, the patients who continued their poor EHs either did not benefit sufficiently from the procedure or, if they did benefit, quickly regained their previous weight after the treatment. This finding is consistent with the literature.

The most important factor affecting patient satisfaction after IGB placement is whether their expectations of weight loss have been met [13,21,22,23]. Psychologically, although the initial weight loss may appear to be a success, the emotional investment in the weight loss process, followed by subsequent weight regain, can lead to dissatisfaction among patients. Another factor contributing to the dissatisfaction resulting from post-procedural weight regain may stem from patients’ expectations that the achieved weight loss would be sustainable and permanent [9,10]. Avcı et al. reported that 51.5% of the patients were satisfied with the procedure based on data obtained at the time of IGB removal [8]. Haddad et al. found that 39% of patients were satisfied when evaluated an average of 3.3 years after balloon removal [14]. In the present study, 45.16% of the patients were satisfied 6 months after IGB removal. The lower satisfaction rate in the present study compared to Avcı et al.’s findings at the time of IGB removal and the higher rate compared to Haddad et al.’s findings 3.3 years after IGB removal could be attributed to the negative impact of weight regain on satisfaction during extended follow-up periods. Mitura et al. evaluated patient satisfaction levels from a different perspective and found that patients with a total body weight loss of 10% or more after IGB were satisfied with the procedure, whereas those with a loss of less than 10% were not satisfied with the procedure [24]. Consistent with this study, in the present study, the patients who were satisfied with the procedure had a total body weight loss of 17.28% at 6 months after IGB removal, exceeding 10%, while those who were not satisfied had a total body weight loss of 8.44%, falling below 10%. This indicates that patient satisfaction is directly related to the amount of weight lost.

A review of the literature suggests that the long-term success of IGB placement can be determined by the interaction of several factors. The key parameters influencing this process include the improvement of eating habits, regular exercise, emotional resilience, effective stress management, and realistic patient expectations [9,16,21]. Patients who adopt realistic expectations and a health-focused approach may perceive the IGB as a more long-term solution, thereby enhancing the sustainability of their weight loss. Considering these factors, providing individualized support and long-term monitoring strategies to patients following IGB placement can enhance success and play a critical role in maintaining weight loss sustainability.

## 5. Limitations

The present study had some limitations that need to be addressed. First, the literature provided limited data on mid-term patient follow-up after IGB treatment, restricting the foundation for a comprehensive discussion of our findings. Second, the sample size of 62 cases limited the generalizability of our results due to the small number of participants. Third, the use of questionnaire-based assessments, particularly regarding patient satisfaction, introduced subjectivity into the results, as it did not account for variations in patients’ expectations and perceptions, educational levels, and socioeconomic factors. Similarly, recall bias should be considered as another limitation, as it can pose a fundamental constraint in survey-based studies. Fourth, the retrospective and single-center design of this study also constituted additional limitations.

## 6. Conclusions

In conclusion, despite the aforementioned limitations, the present study provided valuable insights into the long-term outcomes of IGB treatment and highlighted areas for improvement. In particular, patients who undergo IGB placement for obesity and wish to maintain long-term weight loss should address their poor EHs. Furthermore, the level of patient satisfaction was closely linked to whether their weight loss expectations were met. We believe that future studies should focus on assessments regarding which dietary habit is more effective for sustaining weight loss achieved through IGB placement.

## Figures and Tables

**Table 1:** EH assessment questionnaire.

EHs	Scores
Meal number of times (per day)	
1 times	1 point
2–3 times	2 point
>3 times	3 points
Meal duration (minute)	
>20	1 point
10–20	2 point
Within 10	3 points
Meal time	
Fixed time	1 point
Sometimes irregular	2 point
Irregular	3 points
Overeating (per week)	
0–1	1 point
2–3	2 point
>3	3 points

EH: eating habit.

**Table 2:** Body weight and BMI of patients before and after IGB.

	Before IGB	At the Time of IGB Removal	Six Months After IGB Removal	*p* Value
Body weight (kg)	91.58 ± 7.04	79.16 ± 6.72 ^a^	80.08 ± 7.84 ^b^	<0.001 *
BMI (kg/m^2^)	32.00 (4.25)	28.00 (3.00) ^a^	28.50 (4.25) ^b^	<0.001 ^©^

IGB: intragastric balloon, BMI: body mass index, **^©^** Friedman’s two-way analysis of variance with Bonferroni correction, * repeated measures analysis of variance (ANOVA) with Bonferroni correction, data are presented as mean ± standard deviation for those with normal distribution and median (interquartile range) for those without normal distribution. ^a^ A statistically significant difference is observed between the before IGB and at the time of IGB removal groups, and ^b^ a statistically significant difference is also observed between the before IGB and six months after IGB removal groups.

**Table 3:** Changes in EHTS before IGB and at 6 months after IGB removal for the satisfied and dissatisfied patients.

	Satisfied	Dissatisfied	*p* Value
Pre-IGB EHTS	8.28 ± 1.18	8.02 ± 1.11	0.384 *
EHTS at 6 months after IGB removal	6.82 ± 0.90	7.97 ± 1.29	<0.001 *
*p* value	<0.001 **	0.396 **	

IGB: intragastric balloon, EHTS: eating habit total score, data are presented as mean ± standard deviation, * unpaired samples *t*-test, ** paired samples *t*-test.

**Table 4:** Comparison of the patients who were satisfied and dissatisfied with the IGB.

	Satisfied (n = 28, 45.16%)	Dissatisfied (n = 34, 54.86%)	*p* Value
Age	33.17 ± 9.69	33.29 ± 8.75	0.911 *
Gender (male/female)	9 (36%)/19 (51.35%)	16 (64%)/18 (48.65%)	0.233 ^¥^
% weight loss	17.28 ± 3.28	8.44 ± 3.01	<0.001 ^α,^*
% BMI loss	17.60 ± 3.62	8.47 ± 3.35	<0.001 ^α,^*
Duration of IGB treatment	5.85 ± 0.35	5.82 ± 0.35	0.752
Maximum weight lost during IGB treatment	17.64 ± 3.68	11.41 ± 3.43	<0.001 ^α,^*
Time to reach the maximum weight lost	3.53 ± 0.69	4.23 ± 0.95	0.002 ^α,^*

IGB: intragastric balloon, BMI: body mass index, data are presented as mean ± standard deviation, * Student’s *t*-test, ^¥^ chi-square test, ^α^ bold *p* values indicate significance.

## Data Availability

The original contributions presented in this study are included in the article. Further inquiries can be directed to the corresponding author.
